# Supplementary material for: Production and Characterization of Poly-γ-Glutamic Acid by Bacillus velezensis SDU
Source: Microorganisms. 2025 Apr 16;13(4):917. doi: 10.3390/microorganisms13040917 (PMC12029172; doi:10.3390/microorganisms13040917)
Supplement: Supplementary file 1 [file microorganisms-13-00917-s001.zip › microorganisms-3561008-supplementary.pdf]

Supplementary Material

1 Amino acid of the hydrolysis fermentation products of strain SDU.

Table s1. Amino acid of the hydrolysis fermentation products of strain SDU.

Pk #	RT	Name	Height	Area	ESTD Conc/nmol	Conc/ng
1	5.007	Asp	9126	153771	0.065	8.587
2	5.633	Thr	3379	64154	0.025	2.999
3	6.187	Ser	5538	110670	0.042	4.367
4	7.153	Glu	190270	3787244	1.510	222.181
5	10.007	Gly	3826	70214	0.030	2.239
6	10.920	Ala	7363	187038	0.078	6.966
		Cys			0.000 BDL	0.000
8	12.907	Val	11042	315410	0.121	14.131
		Met			0.000 BDL	0.000
10	16.707	Ile	858	20735	0.008	1.113
		Leu			0.000 BDL	0.000
11	19.273	Tyr	12436	521873	0.209	37.886
12	20.180	Phe	7960	335316	0.113	18.729
14	22.267	Lys	5830	104951	0.035	5.169
15	23.400	NH3	188922	4781262	2.078	35.321
16	24.513	His	1715	52105	0.019	2.948
		Arg			0.000 BDL	0.000

2 GPC-MALLS for molecular weight determination of γ-PGA

Table s2.MW Averages

Peaks	Mp (g/mol)	Mn (g/mol)	Mw (g/mol)	Mz (g/mol)	Mz+1 (g/mol)
Peak 1	1153847	1106091	1167226	1272937	1610081

Table s3.MW Ranges

Peak Number	High Limit MW	Low Limit MW	Percent MW
1	171465301	5000000	0.05
1	5000000	3000000	0.1
1	3000000	1000000	68.09
1	1000000	771294	31.76

2.1 GPC-MALLS Figures

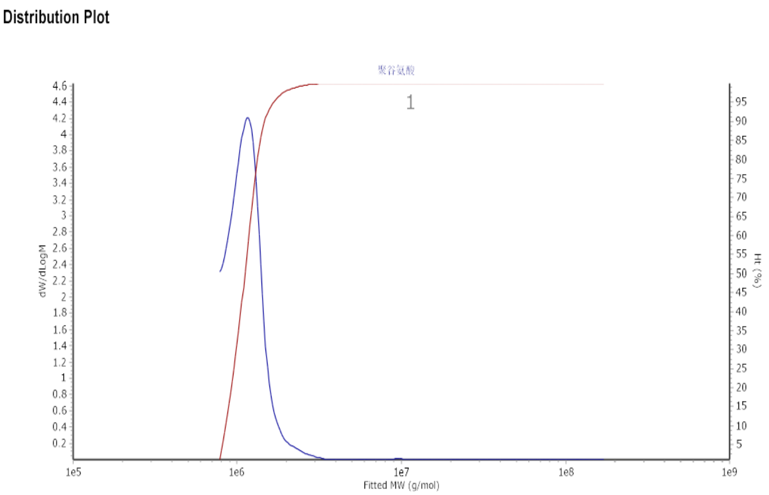

Supplementary Figure s1. GPC-malls results of γ-PGA produced by strain SDU

3 Mark-Houwink equation for molecular weight determination of γ-PGA

Table s4. Mark-Houwink equation for molecular weight determination of γ-PGA

C (g.ml-1)	t（s）	ηr	ηsp	ηsp/C	【η】	M
0.10000	71.90	5.54	4.54	45.39	24.57	1.39×10^{6}
0.07500	49.77	3.83	2.83	37.79		
0.05000	33.78	2.60	1.60	32.05		
0.02500	23.12	1.78	0.78	31.25		

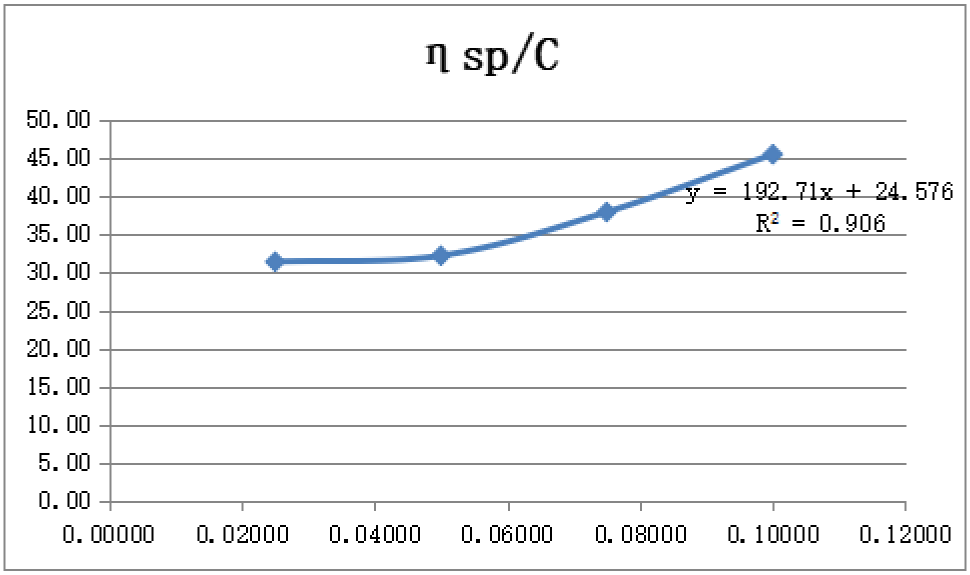

Supplementary Figure s2. Mark-Houwink equation for the value of 【η】
